# Exploration of Prolonged Remission and the Natural Course of Cluster Headache: An Interview-Based Cohort Study

**DOI:** 10.1212/WNL.0000000000213795

**Published:** 2025-06-13

**Authors:** Willemijn Naber, Paulien van Tilborg, Anna Zuidgeest, Leopoldine Wilbrink, Wim Mulleners, Roemer Brandt, Rolf Fronczek

**Affiliations:** 1 Department of Neurology, Leiden University Medical Center, the Netherlands;; 2 Department of Neurology, Zuyderland Medical Center, Heerlen, the Netherlands;; 3 Department of Neurology, The Migraine Clinic, Amsterdam, the Netherlands;; 4 Department of Neurology, Leiden University Medical Center (LUMC), the Netherlands; and; 5 Stichting Epilepsie Instellingen Nederland (SEIN), Sleep-Wake Center, Heemstede, the Netherlands.

## Abstract

**Background and Objectives:**

The aim of this study was to gain rare insight into prolonged cluster headache (CH) remission by (1) identifying patterns and factors associated with and (2) phenotypical changes before prolonged remission. The results can help patients better understand their disease course and uncover mechanisms behind spontaneous remission.

**Methods:**

In this cross-sectional cohort study, all participants with a history of (probable) CH from the Leiden University Medical Center cohort were invited to complete a screening survey. Participants in prolonged remission were invited for a telephone interview. Prolonged remission was defined as (1) no current prophylactic treatment and (2) an attack-free period of ≥5 years and/or twice the mean between-episode time. Main outcomes are average age at prolonged remission onset and disease duration. Data were collected between April 10 and August 9, 2024, and analyzed using descriptive and survival statistics.

**Results:**

Of those invited, 43.2% (778/1,801) responded; 625 were included in the survey analysis, and 125 (20%) met prolonged remission criteria during interview. The median age at inclusion was 58 years (interquartile range [IQR] 48–67) with 32% female. Remission occurred on average at age 55 (IQR 48–63) after a disease duration of 23 (15–33) years. In 62% (N = 78), remission occurred abruptly. Of those with gradual remission (38%, N = 47), attack frequency (65%) and intensity (59%) decreased and between-episode intervals increased (52%) before remission. Probability of prolonged remission was higher in those with episodic CH (hazard ratio [HR] 6.60, 95% CI 3.55–12.31), who had quit smoking (HR 2.53, 95% CI 1.66–3.86), who had a higher attack intensity (HR 1.28, 95% CI 1.08–1.52), and who had a higher age at disease onset (HR 1.05, 95% CI 1.03–1.06).

**Discussion:**

This cohort offers rare insight into prolonged CH remission, typically starting around the mid-50s after 25 years of active disease. Prolonged remission is not tied to a single factor such as disease duration. Remission onset does not peak at a specific age, and disease duration varies widely between patients with remission. Remission probability is higher in the episodic form despite a longer disease duration compared with the chronic form. The association between quitting smoking and prolonged remission supports a causal link with smoking and disease activity. These preliminary retrospective results require confirmation in future studies.

## Introduction

Cluster headache (CH) is a very severe form of headache that is characterized by unilateral headache attacks that are accompanied by various ipsilateral autonomic symptoms or restlessness.^1^ The prevalence of CH in the general population is around 1 in 1,000.^2^

In episodic CH (ECH), the most common form of the disease, the attacks occur in episodes lasting weeks or months, with remission intervals of months to years.^1^ People with chronic CH (CCH, 20% of patients) have headache attacks with less than 3 consecutive months of remission per year.^2^ The chronic form can be unremitting from onset (primary CCH) or can evolve from the episodic form (secondary CCH), and the chronic form can become episodic (secondary ECH).^3,4^ The overall incidence of a subtype switch ranges from 20.7% to 31.6%, with a 5-year risk of 12.3% for conversion into secondary CCH and 25% to secondary ECH.^5,6^ Before transitioning from ECH to CCH, an increase in disease burden is commonly observed. Cluster episodes become longer and attacks are more frequent while the attack-free intervals between episodes shorten.^7^

CH typically starts around age 25–35 years^7-9^ with a higher age at onset in primary CCH compared with primary ECH for both men and women.^10,11^ While some epidemiologic data are available on the onset of CH, little is known about the “end” of CH. The average disease duration and onset age of prolonged remission have not been described in detail. Based on clinical experience, we assume that CH goes into remission with advancing age, mainly substantiated by the fact that few older persons with CH are treated in outpatient headache clinics. Patients are informed that CH is a self-limiting disease that will remit at some point during their lifetime. Earlier small case series report patients whose CH progressively ameliorated and tended to remit with age.^10,12^ Suggested precipitating factors for prolonged CH remission are increased intervals between episodes and episodes with decreasing seasonality, unilaterality of attacks, and fewer autonomic symptoms.^13,14^ However, no predictor of prolonged remission has been found; the occurrence of prolonged remission seems not directly related to older age, longer disease duration, or an accumulation of lifetime episodes.^15^ While the abovementioned supports the hypothesis that CH gradually ameliorates during the disease course, other small studies reported unchanged phenotypes during the course of a follow up period of 3–40 years.^14,16^

The aim of this study was to gain insight into the intriguing yet sparsely documented phenomenon of prolonged remission in CH. We aim to identify characteristics, patterns, and factors associated with prolonged remission as well as phenotypical changes before remission in CH. As a result, we can provide patients with more accurate information regarding their potential disease course. Furthermore, by increasing our understanding of spontaneous remission in CH, we hope to uncover potential underlying mechanisms that can help therapeutic research to instigate therapeutic prolonged remission in people with active CH.

## Methods

### Design and Participants

In this single-center observational cross-sectional study, all participants from the Leiden University Medical Center-Cluster Headache cohort (LUMC-CH) were invited by email to complete an online screening survey. The LUMC-CH consists of the Leiden University Cluster headache neuro-Analysis cohort, which is a validated, web-based cohort developed in 2008 with a screening questionnaire for CH based on the ICHD criteria.^17^ This screening questionnaire has been continuously updated to align with the latest ICHD criteria. This cohort is supplemented with patients from our outpatient headache clinic with a neurologist-confirmed CH diagnosis according to the International Classification of Headache Disorders, third edition (ICHD-3) criteria.^17^ To ensure alignment with the most recent classification standards, all participants—regardless of initial inclusion under ICHD-2 in 2008—are re-screened against the current ICHD-3 criteria before inclusion. Inclusion criteria for this study were as follows: (history of) having (probable) CH as defined by the ICHD-3 criteria,^1^ age 16 years or older, and proficiently fluent in Dutch to complete the screening questionnaire.

The invitation email contained a digital patient information form (eAppendix 1) with the outline of the study and a direct link to the online screening survey, distributed through Castor Electronic Data Capture. Participants were informed that this study would focus on the course of their CH symptoms, and patients with and without active CH were recruited. Nonresponders received 4 reminder emails, each containing the patient information form, to boost the response rate.

The online survey was designed as a screening tool for prolonged remission. In a recent study on the natural course of untreated CH, remission was defined as being attack-free for longer than twice the *longest* between-episode period *and* for at least 5 years.^13^ However, duration of the between-episode period relies on patients' recollection and may be subject to bias. In routine practice, the *average* between-episode period may better reflect the duration of the symptom-free interictum, as it is easier to estimate than recalling the longest between-episode period. In addition, a more lenient definition of prolonged remission was more appropriate for this study because it was important not to miss any cases during screening. Therefore, we defined prolonged remission as (1) no current CH prophylactic treatment and (2) an attack-free period of either ≥5 years *and/or* twice the *mean* between-episode time.

All eligible patients who screened positive for prolonged CH remission were invited for a telephone interview. In this interview, phenotypical changes in relation to prolonged remission were further explored in a standardized and semistructured manner. All interviews were performed by a CH specialist (W.N.) and 2 purpose-specific trained medical students (M.G., A.Z.), who consulted with the specialist in cases of ambiguity. Medical terms such as “episodic” and “chronic” CH and “remission” were explained during the interview. The estimated time of interview completion was 20 minutes. Survey and interview questions are presented in eAppendix 2.

### Standard Protocol Approvals, Registrations, and Participant Consents

Because this study only involved a nonburdensome questionnaire, the provided informed consent was sufficient for study participation according to the Dutch medical research law (Medical Research Involving Human Subjects Act). Therefore, an exemption for additional medical ethical review was provided by the Medical Research Ethics Committee of the LUMC (METC-LDD; reference number 24-3020). Data were collected between April 10 and August 9, 2024.

### Statistical Analysis

The main outcomes are the average age at prolonged remission onset and the years with active CH until prolonged remission. Onset of prolonged remission is defined as the first day of the attack-free period (the day after the last CH attack). Disease duration is the amount of years from CH onset until prolonged remission onset or inclusion. Sensitivity analyses were performed to determine (1) whether participants with *lenient* prolonged remission (attack free for ≥5 years *or* twice the mean between-episode time) differed significantly from those with *strict* prolonged remission (attack free for ≥5 years *and* twice the mean between-episode time) and (2) whether participants who met the ICHD-3 criteria for probable CH differed from those who met criteria for definite CH (ICHD-3 criteria: *3.5.1 probable cluster headache = missing one of the features required to fulfil all cluster headache criteria*).

All the descriptive data are presented as number (percentage) or median (interquartile range), unless otherwise specified. For group comparisons, χ^2^ tests, Student *t* tests, or Mann-Whitney *U* tests were performed when appropriate. A survival analysis was applied to determine the influence of CH characteristics on the probability of prolonged remission in the population who completed the screening survey (both participants with and without prolonged remission). Time of interest is the number of years since CH onset, as a measure of disease duration. The event of interest was onset of prolonged remission. Participants who completed the survey without having prolonged remission were censored at the time of survey completion. A Kaplan-Meier curve with a log-rank test was plotted to determine overall probability of prolonged remission as stratified for ECH and CCH. A Cox proportional hazard model was fitted to extract hazard ratios (HRs) for each of the CH characteristics (e.g., attack frequency and sex) to assess their impact on the probability of prolonged remission.

Two-tailed *p* values less than 0.05 were considered statistically significant. No correction for multiple testing was applied because of the exploratory nature of our research. All analyses were performed using RStudio (R Foundation for Statistical Computing, Vienna, Austria), version 4.3.1, and the *survminer* and *survival* packages were used for survival analysis.

### Data Availability

The data sets used and/or analyzed during the present study are available from the corresponding author on reasonable request.

## Results

### Participants

**Figure 1:**
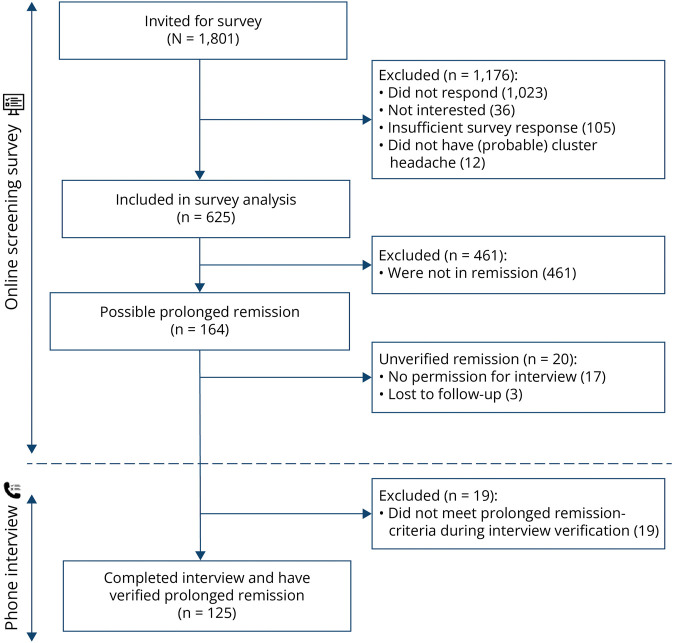
Flowchart of Inclusion Process A total of 625 of 1,801 invited patients with cluster headache completed the screening survey and fulfilled the inclusion criteria. One hundred sixty-four participants screened positive for possible prolonged remission, and of those, 125 met remission criteria during interview and were included in analysis.

As shown in Figure 1, 778 of 1,801 invited persons (43.2%) responded to the screening survey and 625 fulfilled the inclusion criteria. One hundred sixty-four participants screened positive for possible prolonged remission in the survey, and of those, 125 met remission criteria during interview and were included in prolonged remission analyses. Nonresponders (N = 1,023) were on average younger (53 vs 60 years old) and more often female compared with responders (37% vs 32%); subtype distribution was equal to that of responders (58% had ECH, eTable 1).

Clinical characteristics are presented in Table 1. Most of the 625 included participants had ECH (372 [60%]) and were male (male-to-female ratio of 2.1:1) with an average age at inclusion of 58 (48–67) years. No clinical differences were found between participants with probable CH and those with definite CH. One participant experienced a single 6-week cluster episode and, while meeting ICHD-3 criteria for CH, was classified as having probable ECH because of the absence of a second episode over an 11-year follow-up. For readability, this participant was analyzed as having the episodic subtype, as exploratory analysis confirmed that inclusion did not affect the main outcomes.

**Table 1:** Baseline Characteristics of Participants

	Total (N = 625)	No remission (N = 480)^a^	Prolonged remission (N = 125)	*p* Value
Interviewed	144 (23)	19 (4)	125 (100)	
Prolonged remission^b^	125 (23)	0 (0)	125 (100)	
Attack free, y	0.5 (0.2–3.1)	0.2 (0.2–0.9)	6 (4–11)	<0.001^d^
Active CH^c^	379 (61)	246 (51)	0 (0)	<0.001^d^
Prophylactic use	257 (41)	257 (54)	0 (0)	<0.001^d^
Sex				0.003^d^
Male	424 (68)	310 (65)	98 (78)	
Female	201 (32)	170 (35)	27 (22)	
Age at inclusion	58 (48–67)	57 (46–65)	64 (55–70)	<0.001^d^
Age at CH onset	29 (20–42)	29 (20–43)	27 (19–39)	0.120
Age at menarche	13 (12–14)	13 (11–14)	14 (12–14)	0.130
Age at menopause	49 (45–51)	49 (45–51)	51 (49–55)	0.023^d^
Disease duration	21 (13–31)	20 (13–30)	23 (15–33)	0.053
Episodic CH^c^	372 (60)	240 (50)	113 (90)	<0.001^d^
Subtype switch	167 (27)	146 (30)	21 (17)	<0.001^d^
Attack frequency	3 (1–4)	3 (1–4)	3 (1–5)	0.600
Attack intensity	9 (8–10)	9 (8–10)	9 (9–10)	<0.001^d^
Autonomic symptoms	583 (94)	456 (95)	111 (90)	0.017^d^
Restlessness	562 (90)	431 (90)	113 (90)	0.900
Episode duration, wk	8 (5–13)	8 (6–13)	8 (5–12)	0.300
Between-episode interval, mo	9 (5–12)	9 (6–18)	9 (4–12)	0.058
Comorbid headache	178 (29)	140 (29)	31 (25)	0.500
Smoker	165 (27)	143 (30)	17 (14)	<0.001^d^
Positive family history	67 (11)	47 (9.8)	17 (14)	0.200
Right-sided attacks	256 (41)	198 (41)	47 (38)	0.500
Bilateral attacks	12 (1.9)	8 (1.7)	3 (2.4)	0.700

Abbreviations: CH = cluster headache; IQR = interquartile range.

Descriptives are depicted as median (IQR) or n (%).

a Twenty of 625 screened positive for remission in the questionnaire, but criteria could not be verified because of unavailability for phone interview. These 20 patients were, therefore, excluded from group comparison (remission vs no remission), resulting in N = 480 participants without remission (and not 625–125 = 500).

b Verified prolonged remission based on the following: screened positive in the survey and remission criteria were confirmed during the telephone interview.

c Active CH: having CH attacks in the month of inclusion.

d Statistically significant result.

**Figure 2:**
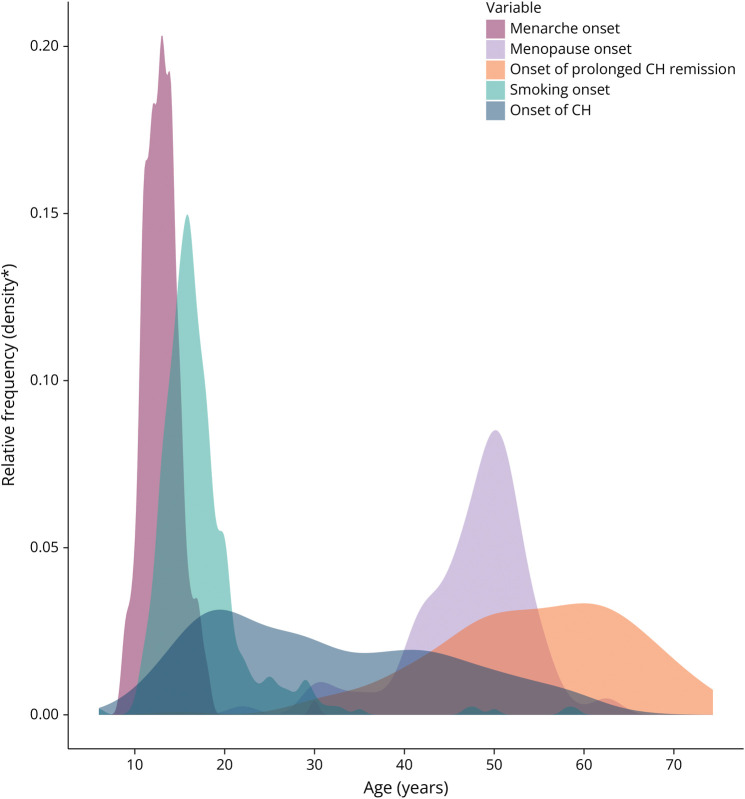
Density Plot Depicting the Distribution of Age at Onset This figure illustrates the age distribution for the following events within this CH cohort: (1) onset of CH, (2) onset of prolonged remission, (3) onset of smoking, (4) menarche, and (5) menopause in this CH cohort. CH = cluster headache.

The median age at CH onset was 29 years (20–42) (male: 30 [20–42]; female: 27 [19–43]; ECH: 26 [19–40]; CCH: 34 [23–45]). The distribution of the age at onset is displayed in Figure 2. No correlation between the age at smoking onset and age at menarche and the start of CH was found. The average diagnostic delay was 3 (1–8) years.

In participants with ECH, 88.5% had an average interval of 2.5 years or less between episodes and 97.1% had less than 5 years between episodes (eFigure 1). The median interval between episodes was 10 months.

### Prolonged Remission

#### Participants

Prolonged remission was verified in 20% (N = 125/625) of participants. Of those 125, 113 (90%) had ECH and 12 (10%) had CCH (Table 1). The prolonged remission cohort had a male-to-female ratio of 3.6:1, and the average age at inclusion of 64 (55–70) years. No differences between participants meeting the strict or lenient prolonged remission criteria were observed.

#### Remission Disease Course

**Figure 3:**
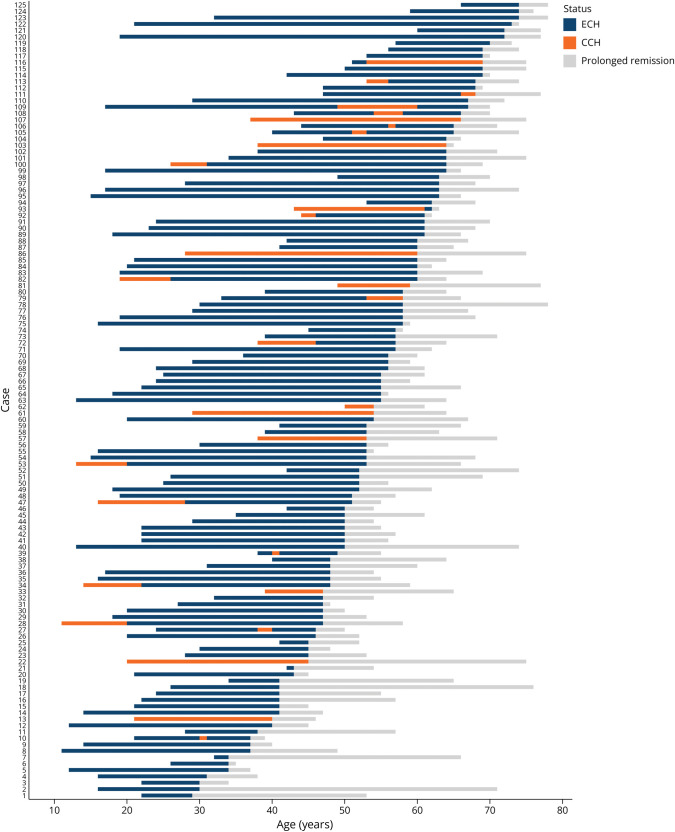
Disease Course of Interviewed Participants With Prolonged Remission This figure illustrates the disease course of all 125 interviewed prolonged remission participants. The disease courses are displayed as a bar for each participant, which indicates the duration between the age at CH onset and age at prolonged remission onset in blue and/or orange (periods with ECH in orange, periods with CCH in blue) and the duration between age at prolonged remission onset and age at inclusion in gray. CCH = chronic CH; CH = cluster headache; ECH = episodic CH.

The disease course of prolonged remission is presented in Figure 3. The median age at prolonged remission onset was 55 years (48–63) (male: 56 [48–63]; female: 50 [45–64]; ECH: 55 [48–63]; CCH: 54 [47–60]). On average, CH lasted 23 (15–33) years before prolonged remission (male: 25 [17–35]; female: 17 [13–29]; ECH: 26 [16–35]; CCH: 18 [10–25]).

A subtype change during their disease course was reported by 21 (17%). Thirty interview participants met CCH criteria at some point during the course of their disease, of whom 20 (67%) had primary CCH. At the end of their CH before prolonged remission, 40% (N = 12) still had CCH and 60% (N = 18) had switched to ECH. Twelve (86%) had a temporal side shift, and in only 2, this was permanent. The shift occurred during an episode in 46% (N = 6) and between episodes in 54% (N = 7).

#### Prolonged Remission Phenotype

**Figure 4:**
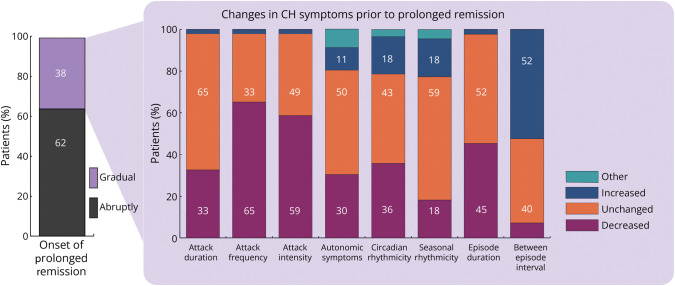
Phenotypical Changes Before Prolonged Remission Prolonged remission occurred abruptly in 62% (N = 78). Of those with gradual prolonged remission (N = 47 [38%]), attack frequency (65%) and intensity (59%) decreased and between-episode intervals increased (52%) in most participants before prolonged remission. CH = cluster headache.

Phenotypical patterns of prolonged remission are presented in Figure 4. Prolonged remission occurred abruptly in 62% (N = 78). Of those with gradual prolonged remission (N = 47, 38%), attack frequency (65%) and intensity (59%) decreased and between-episode intervals increased (52%) in most participants before prolonged remission. Circadian rhythmicity over the years was unchanged in 43%, more predictable in 18%, and less predictable in 36%. For seasonal rhythmicity, this was unchanged over the years in 59%, more predictable in 18%, and less predictable in 18%.

#### Self-Reported Triggers of Prolonged Remission

Most participants with prolonged remission (N = 72 [57.6%]) did not report any events before the onset of prolonged remission that might have triggered amelioration of CH symptoms. Of the self-reported categorized triggers, alternative treatments (N = 18 [13.6%]), lifestyle changes (N = 17 [13.6%]), and quitting smoking (N = 8 [6.2%]) were mentioned most frequently (eFigure 2). Most self-reported alternative treatments were metoprolol (N = 4), orthopaedic manual therapy (N = 4), and illicit drugs (N = 4). Lifestyle changes mostly consisted of reduced stress (N = 12) and quitting alcohol consumption (N = 2).

#### Remission Determinants

**Figure 5:**
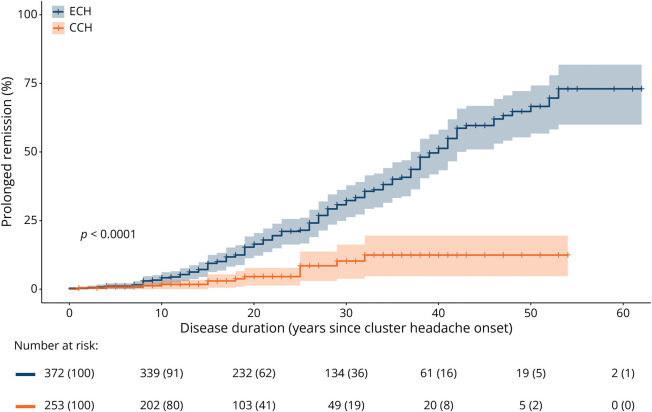
Kaplan-Meier Curve With 95% CIs of Prolonged Remission A Kaplan-Meier curve depicting the probability and 95% CIs of prolonged remission in relation to disease duration split for the ECH and CCH. The log-rank test shows that the ECH group has a shorter disease duration when reaching prolonged remission (HR 0.15, 95% CI 0.08–0.28, *p* < 0.001). CCH = chronic CH; CH = cluster headache; ECH = episodic CH; HR = hazard ratio.

CH subtype had a significant effect (HR 6.60, 95% CI 3.55–12.31, *p* < 0.001) on the probability of prolonged remission, as shown in Figure 5. All covariates in the fitted Cox proportional hazard model met the proportionality assumption (eFigure 3). In addition to having ECH, participants who had quit smoking had a shorter disease duration before prolonged remission compared with current smokers (HR 2.53, 95% CI 1.66–3.86, *p* < 0.001) (Table 2). A higher probability of prolonged remission was also observed in participants with a higher attack intensity and higher age at onset of CH (attack intensity: HR 1.28, 95% CI 1.08–1.52, *p* = 0.004; age at onset: HR 1.05, 95% CI 1.03–1.06, *p* < 0.001).

**Table 2:** Cox Proportional Hazard Model for Prolonged Remission

	β	HR (95% CI)	*p* Value
Subtype, ECH	1.888	6.60 (3.55–12.31)	<0.001^a^
Sex, male	−0.107	0.90 (0.60–1.34)	0.597
Sex, female	0.107	1.11 (0.75–1.66)	0.597
Age at onset, y	0.047	1.05 (1.03–1.06)	<0.001^a^
Attack frequency, daily	−0.009	0.99 (0.93–1.06)	0.782
Autonomic symptoms, yes	−0.400	0.67 (0.39–1.16)	0.156
Attack intensity, NRS score	0.246	1.28 (1.08–1.52)	0.004^a^
Restlessness, yes	0.275	1.32 (0.73–2.39)	0.365
Comorbid headache, yes	−0.246	0.78 (0.53–1.14)	0.204
Smoking, never	0.101	1.11 (0.74–1.65)	0.620
Smoking, quit smoking	0.930	2.53 (1.66–3.86)	<0.001^a^
Family history, first grade	0.304	1.36 (0.85–2.15)	0.197
Family history, second grade	−0.474	0.62 (0.20–1.98)	0.421

Abbreviations: β = estimated regression coefficient; ECH = episodic cluster headache; HR = hazard ratio; NRS = Numeric Rating Scale.

a Statistically significant result.

## Discussion

This cross-sectional observational study provides a comprehensive insight into prolonged remission of CH. Prolonged remission was confirmed in 20% of this cohort. On average, prolonged remission of CH starts at age 55 (48–63) after a disease duration of 23 (15–33) years. Our data did not identify 1 unique pattern preceding prolonged remission. While most reported an abrupt onset of prolonged remission, gradual onset was present in almost 40%. When prolonged remission started gradually, this was characterized by decreased frequency and intensity of cluster attacks and lengthening intervals between episodes before prolonged remission. No common trigger for prolonged remission was reported. In this cohort, patients with ECH who had a higher attack intensity, a higher age at CH onset, and quit smoking had a shorter disease duration before prolonged remission was reached.

In our cohort, almost 90% of intervals between episodes are shorter than 2.5 years, which supports the suitability of our scientific definition for prolonged remission (attack free for ≥5 years or twice the mean between-episode time, eFigure 4).

Our findings suggest that prolonged remission is not a direct effect of age or longer disease duration and the clinical observation that prolonged remission seems to be more common in older patients can likely be attributed to cumulative risk. We observed a considerable variation in disease duration until remission, ranging from 6 weeks to 55 years. In addition, the distribution of the age at remission onset does not exhibit a peak at a specific age but instead follows a broad distribution, with the youngest participant achieving remission at 15 and the oldest at 74. Finally, patients with ECH have a higher likelihood of remission despite its longer disease durations compared with those with CCH. Neither selection bias nor differences in prolonged remission criteria between ECH and CCH could explain the high prevalence of episodic patients experiencing prolonged remission, suggesting that this trend is not caused by the study design but may be a feature of prolonged remission. These findings imply that prolonged remission is not merely a result of advancing age or disease duration but rather the result of a complex multifactorial process.

A possible model to explain why prolonged remission is more common in the episodic subtype is the threshold-based hypothesis.^18^ This model suggests that CH has a fluctuating disease process that causes CH attacks only when the disease activity surpasses a predetermined threshold. We hypothesize that the disease activity is more often below the CH threshold in ECH than in CCH, resulting in remission periods. Prolonged (and possibly permanent) remission could then very well be explained as an extended period below the CH threshold. Of interest, in most of the participants who had CCH at any time during their disease course, their CH converted to ECH before reaching prolonged remission, suggesting a gradual decrease in their disease activity. We hypothesize that this decreased disease activity is a consequence of reduced hypothalamic activity through a complex interplay of multiple factors (e.g., changes in connectivity to the suprachiasmatic nucleus, epigenetics, and fluctuations in vasoactive peptides and [sex] hormones).

The observed age at remission onset in CH is similar to the reported prevalence patterns in migraine, where remission also occurred around 50 years of age.^19^ In migraine, age-related prevalence fluctuations are often attributed to hormonal fluctuations that occur with menarche or menopause.^19^ However, despite some similarities, no biphasic peak around menarche or menopause was observed, nor were these events strongly correlated with the onset or remission of CH, making a direct causal link between hormonal fluctuations and prolonged remission in CH less likely.

Our results suggest that disease duration until prolonged remission was shorter in patients who had quit smoking, whereas previously, no factors that were associated with prolonged remission were found.^13^ A causal relationship between smoking and CH was suggested by a Mendelian randomization study.^20^ It is thus interesting that quitting smoking was associated with a higher probability of prolonged remission. Quitting smoking may be one of the factors altering epigenetics, leading to reduced disease activity. However, causality remains unclear because our model only identifies correlation. Quitting smoking was only reported by 6.2% as remission trigger, and visual data exploration did not show an obvious relationship between the timing of quitting smoking and onset of prolonged remission.

In comparison with earlier studies, in our cohort, prolonged remission occurred when patients were older and had a longer disease duration, despite having a similar age at onset of CH.^13,14,21^ Contrary to our observations in the outpatient headache clinic, our results indicate that older individuals with a long disease duration can still have active CH. Perhaps, the lack of older patients in the outpatient clinic is not caused by a lack of disease, but the fact that “experienced” CH patients with a long disease duration no longer consult a doctor at recurrence of headache attacks because they are familiar with the pain and possible treatments.^22^

The observation that prolonged remission is not preceded by 1 unique pattern but can occur abruptly or gradually likely explains the discrepancy between reports that describe unchanged phenotypes during the disease course on one hand and improving CH symptoms before prolonged remission on the other hand.^13,14,16,23^ In some of our patients, the disease course remained unchanged for decades, while in others, CH symptoms gradually ameliorated. In addition to the previously reported observation of lengthening between-episode interval without shortening of the episodes before prolonged remission, we also observed a decrease in attack frequency and intensity.^13,14^

The Kaplan-Meier curve (Figure 5) shows a slower remission rate than might be expected when compared with the median disease duration of 23 years in participants with remission. The discrepancy is caused by the methodological differences between survival analyses and descriptive statistics. The Kaplan-Meier analysis includes the total population, resulting in a reduced sample size at the end of follow-up because the total follow-up duration (CH onset until remission or inclusion) was not the same for all participants. It uses censoring to account for the possibility of patients whose CH will remit in the future, and it does not assume that all patients will eventually go into remission but represents the probability of remission over time for the entire population, including those who may never achieve it. Therefore, the discrepancy between the descriptive statistic and survival analysis is likely due to a combination of factors: differences in methodology and, therefore, interpretation; a decreasing sample size with longer follow-up; and the unanswered question of whether all patients with CH will eventually achieve remission.

With this large and well-described cohort, we provided an unique insight into the phenotype of prolonged remission in CH. Previous studies were small and mostly consisted of cohorts of patients who dropped out of clinical care. A major strength of this study is its detailed approach that focuses on the different aspects of prolonged remission. This results in a comprehensive description of both CH characteristics and phenotypical changes in relation to prolonged remission. Data are of high quality as they are collected in a standardized and systematic manner using a screening survey followed by a structured interview and by posing the more complicated questions during a telephone interview instead of the online survey.

The study design also has some limitations. Because a prospective design was not feasible because of the long disease duration, a retrospective design was chosen with the acknowledged limitation of an increased risk of recall bias. The study design incorporated precautions to minimize recall bias: (1) detailed questions were addressed during the interview and (2) mainly closed-ended questions were used. In addition, comorbidities were not included in the survey. We aimed to keep the survey concise to ensure reliable responses, and at the time of study development, no specific comorbidities were known to directly influence the onset or course of CH. However, the findings from a previous study^24^ suggest that certain comorbidities (e.g., cancer and cardiovascular disease) may be more common in people with CH compared with the general population, possibly due to an unhealthier lifestyle (higher rates of smoking and alcohol consumption). Future studies should further explore the role of comorbidities in CH, particularly in relation to disease onset and progression, to enhance our understanding of their potential impact. Finally, the inclusion of 1 participant with probable ECH could be considered a potential limitation. However, exploratory analyses comparing results with and without this participant revealed no differences, suggesting that this inclusion did not affect the main outcomes of this study.

Because the study aimed to identify as many patients with prolonged remission as possible, recruitment was not limited to patients with active CH, resulting in a lower response rate (43%) and a selection bias. While the selection bias may have led to an overestimation of the prevalence of prolonged remission (20%) and a higher age in this cohort compared with the general CH population (60 vs 53 years old), this also served to enhance the comprehensiveness of our cohort. Compared with the historical general CH population, our study has a relatively high proportion of women (2.1:1 ratio, compared with the historical reported ratios of 3–4:1). This aligns with the currently reported shift in sex ratios, which report similar ratios of 2:1.^25^ Because sex did not seem to significantly affect remission, the consequences reduced sex ratio appear minimal. Epidemiologic data of this study should not be applied to the general CH population, but the qualitative data should serve as a starting point for further research.

In contrast to migraine, no official definition of prolonged or permanent remission exists in the ICHD-3 criteria.^1^ In this study, we defined our own criteria for prolonged remission based on our clinical experience and previous studies where attack freedom for more than 4 or 5 years was seen as prolonged remission.^13,14,26^ Prolonged remission is not the same as permanent remission because there is no guarantee that patients are in permanent remission and thus “cured” from their CH. There are known cases in which a new cluster episode occurred after decades of remission. However, we confirm that the probability of CH symptom recurrence steeply decreases after 2.5 years.^22^ It seems likely that the described cohort is representative of the phenotype of prolonged remission in CH. We, therefore, propose the following scientific criteria to define prolonged CH remission: attack free for ≥5 years or twice the mean between-episode time.

In conclusion, this cohort provides a rare insight into prolonged CH remission and shows (1) an average age of 30 at CH onset followed by (2) 23 years of active CH before (3) prolonged remission onset when patients reach their mid-50s (eFigure 5). Possible precipitating signs of prolonged remission were decreasing attack frequency and intensity or lengthening between-episode intervals. This study's definition for prolonged remission (attack free for ≥5 years or twice the mean between-episode time) was verified as suitable for future research. Prolonged remission seems complex and cannot be attributed to a single factor such as long disease duration. The distribution of remission onset does not peak at a specific age, and disease duration differs greatly between patients. Remission probability is higher in the episodic form despite a longer disease duration compared with the chronic form. The association between quitting smoking and prolonged remission supports a causal link with smoking and disease activity. These preliminary and retrospective results require confirmation in future studies.

## Glossary

CCH: chronic CH
CH: cluster headache
ECH: episodic CH
HR: hazard ratio
ICHD-3: International Classification of Headache Disorders, third edition
IQR: interquartile range
LUMC-CH: Leiden University Medical Center-Cluster Headache cohort
